# Anticipatory countering of motor challenges by premovement activation of orexin neurons

**DOI:** 10.1093/pnasnexus/pgac240

**Published:** 2022-10-25

**Authors:** Dane Donegan, Daria Peleg-Raibstein, Olivier Lambercy, Denis Burdakov

**Affiliations:** Department of Health Sciences and Technology, Swiss Federal Institute of Technology - ETH Zürich, Schorenstrasse 16, 8603 Schwerzenbach, Switzerland; Department of Health Sciences and Technology, Swiss Federal Institute of Technology - ETH Zürich, Schorenstrasse 16, 8603 Schwerzenbach, Switzerland; Department of Health Sciences and Technology, Swiss Federal Institute of Technology - ETH Zürich, Schorenstrasse 16, 8603 Schwerzenbach, Switzerland; Department of Health Sciences and Technology, Swiss Federal Institute of Technology - ETH Zürich, Schorenstrasse 16, 8603 Schwerzenbach, Switzerland

**Keywords:** motor control, skilled movements, hypothalamus, predictive control

## Abstract

Countering upcoming challenges with anticipatory movements is a fundamental function of the brain, whose neural implementations remain poorly defined. Recently, premovement neural activation was found outside canonical premotor areas, in the hypothalamic hypocretin/orexin neurons (HONs). The purpose of this hypothalamic activation is unknown. By studying precisely defined mouse–robot interactions, here we show that the premovement HON activity correlates with experience-dependent emergence of anticipatory movements that counter imminent motor challenges. Through targeted, bidirectional optogenetic interference, we demonstrate that the premovement HON activation governs the anticipatory movements. These findings advance our understanding of the behavioral and cognitive impact of temporally defined HON signals and may provide important insights into healthy adaptive movements.

Significance Statement Anticipatory movements proactively oppose predictable challenges. In mammals, these vital cognitive and motor actions involve the brain, but the necessary brain components remain incompletely identified. We found that selective disruption of anticipatory movements results from inactivity of neurons not previously implicated in this phenomenon: hypothalamic hypocretin/orexin neurons (HONs). We show that the brain responds to predictable motor challenges by upregulating HON activity, and this neural adaptation is critical for normal anticipatory movements. These findings identify natural neural signals important for anticipatory actions. This may also have clinical implications, since HON inactivity causes narcoleptic inability to move in response to predictable challenges in about 1:1,000 people. Our anticipatory hypothesis of HONs now offers a new perspective on this poorly understood symptom.

## Introduction

Brain systems that counter upcoming challenges with anticipatory actions have long been at the forefront of research across diverse academic fields. Studies in neuroscience, artificial intelligence, and engineering proposed general and neural algorithms for anticipation, and a role of premovement cortical activity in anticipatory actions (1–4). Premovement neural activity refers to neural signals that appear during seconds/subseconds before movement onset, and predict movement initiation. Such signals have been documented in the cortex, basal ganglia, and the lateral hypothalamus (LH) (5–7). The recently described premovement activation of hypothalamic hypocretin/orexin neurons (HONs) (7) is of particular interest, because its purpose in the context of anticipatory movements is unknown.

## Results

**Fig. 1.**
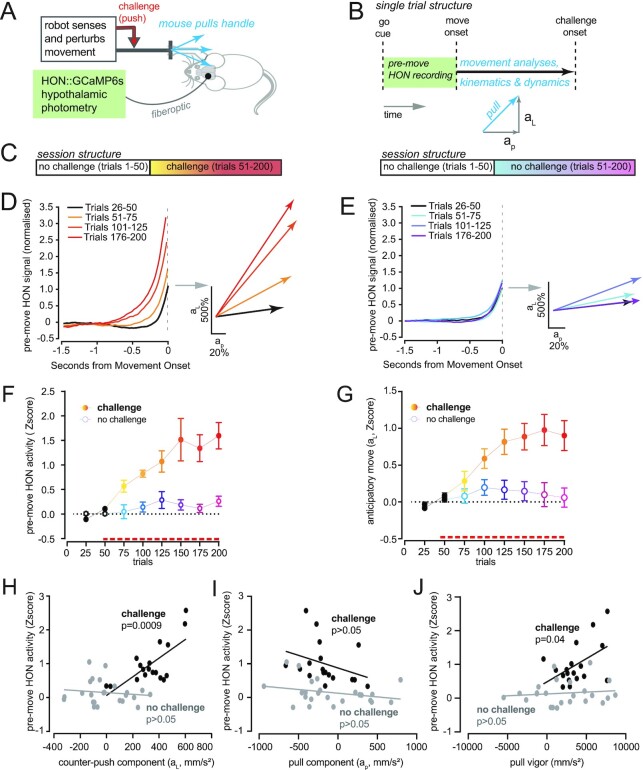
Correlating premovement HON activity and anticipatory movements. (A) Setup diagram (see “Methods” for description). (B) Single trial structure. HON signals are analyzed between the go cue and movement initiation. (C) Session structure, with challenge-containing trials (left) and without (right). (D) Left, mean premovement HON-GCaMP6s activity ( *n* = 15 challenge sessions from four mice). Right, corresponding prechallenge acceleration vectors. (E) Same as (D), but without challenge (*n* = 9 sessions from four mice). (F) HON-GCaMP6s signal amplitude across challenge sessions (*n* = 15 sessions from four mice) and no-challenge sessions (*n* = 9 sessions from four mice). Red dashed line indicates the start of the challenge-containing trails (or not challenge control). 2-way RM ANOVA, challenge F(1, 22) = 14.99, *P* < 0.001; challenge x trials F(7,154) = 4.439, *P* < 0.001). (G) Same as (A), for anticipatory movements (challenge, *n* = 38 sessions from 12 mice; no challenge, *n* = 32 sessions from 12 mice). 2-way RM ANOVA, challenge F(1,102) = 13.99, *P* < 0.001; challenge x trials F(7,714) = 11.59, *P* < 0.0001). (H) Relationship between premovement HON-GCaMP6s signals and corresponding anticipatory counter movement (challenge-opposing acceleration component) with challenge (black) and without challenge (gray). Each point is the average trial epoch (block of 25) for each mouse during trials 50 to 200. Challenge *n* = 18 trial blocks from three mice, Pearson's correlation: r^2^ = 0.506, *P* = 0.0009. No challenge *n* = 24 trial blocks from four mice, Pearson's correlation: r^2^ = 0.016, *P* = 0.557, ns: *P* > 0.05. I. Same as in (H), but for challenge-perpendicular acceleration component. Challenge *n* = 18 trial blocks from three mice, Pearson's correlation: r^2^ = 0.106, *P* = 0.186, ns: *P* > 0.05. No challenge *n* = 24 trial blocks from four mice, Pearson's correlation: r^2^ = 0.078, *P* = 0.186, ns: *P* > 0.05. (J) Same as in (H), but for complete acceleration vector. Challenge *n* = 18 trial blocks from three mice, Pearson's correlation: r^2^ = 0.237, *P* = 0.0040. No challenge *n* = 24 trial blocks from four mice, Pearson's correlation: r^2^ = 0.014, *P* = 0.580.

To examine the role of premovement HON activation in anticipatory movements, we first fiberoptically recorded HON-GCaMP (7) signals in mice trained to pull on a robot-controlled handle (8), where a straight movement (pull) was rewarded (Fig. 1A–C; see “Methods”). To create a challenge during the movement, we programmed the robot to apply a lateral force after a specific point in each pull trajectory, pushing the handle sideways (Fig. 1A). The robot sensed handle position at a high temporal resolution (8). This enabled us to quantify dynamics and kinematics of movements happening before the challenge onset, where challenge-anticipatory movements occur (Fig. 1B and C). Mice adapted to the push challenge by preemptively accelerating in the opposite direction (Fig. 1D). Henceforth, we refer to this as “anticipatory movement” (quantified as acceleration in the opposite direction to the force challenge), because it preceded the challenge, and was not seen in sessions without the challenge (Fig. 1E).

Stronger anticipatory movements were associated with increased premovement HON-GCaMP signals (Fig. 1D). Importantly, in sessions without the push challenge, no such changes in HON activation and movement occurred (Fig. 1E). Statistical quantification revealed that the premovement HON-GCaMP signals stayed steady during sessions without the challenge, but progressively increased across the challenge trials with a similar time-course as the anticipatory movements (Fig. 1F and G). During the challenge trials, the premovement HON activation was significantly correlated with the anticipatory movement component that directionally opposed the push challenge (Fig. 1H, black). This correlation was not observed in the absence of the challenge (Fig. 1H, gray), or in the other (challenge-perpendicular) component of anticipatory movement (Fig. 1I), confirming that it reflects challenge-anticipatory counter action. At the level of the whole vector of the initial pull, we also observed a correlation with HON activity in the presence but not in the absence of challenge (Fig.   1J), as expected from the individual components of this vector (Fig. 1H and I).

**Fig. 2.**
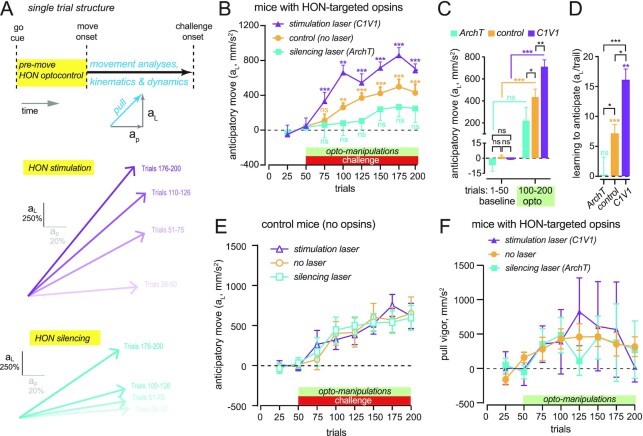
Causal roles of premovement HON signals in subsequent movements. (A) Top, temporal targeting of HON optogenetic interference. Middle, averages of the acceleration vectors of HON optostimulation (orexin::C1V1) sessions in baseline (trials 26 to 50), early (trials 51 to 75), middle (trials 101 to 125), and late (trials 176 to 200) epochs ( *n* = 4 mice). Acceleration vectors are normalized to the magnitude of both acceleration components (*x* and *y*) of baseline trials 26 to 50. Bottom, same as (B), but for HON- optosilencing (orexin::ArchT, *n* = 4 mice). (B) Group data of anticipatory movements during HON optostimulation (magenta, orexin:: C1V1, *n* = 4 mice), HON optosilencing (cyan, orexin::ArchT, *n* = 4 mice), and control (orange, laser off, *n* = 11 mice). (C) Same as (B), but comparing prechallenge (trials 1 to 50) trial averages with during-challenge (trials 100 to 200) trial averages. Data are means ± SEM. Indicated statistics are from Holm–Sidak’s multiple comparison tests (ns = *P* > 0.05, *P < 0.05,**P < 0.01, ***P < 0.001) following 2-way RM ANOVA, group F(2, 15) = 5.662, *P* < 0.05. (D) Same as (B), but comparing slopes of linear fits to the relationship between anticipatory movement and trial number during the first 50 trials of challenge (a metric of learning to anticipate across trials). Data are means ± SEM. Indicated statistics are from Holm–Sidak’s multiple comparison tests (ns =   *P* > 0.05, *P < 0.05,**P < 0.01, ***P < 0.001) following 1-way ANOVA, F(2, 16) = 2.774, *P* < 0.01. (E) Control experiment for (B). Data are means ± SEM of *n* = 8/10/9 sessions from six mice for laser ON (excitation light pattern)/laser OFF/laser ON (silencing light pattern). 2-way RM ANOVA, F(92, 24) = 0.01039, *P* = 0.9897. (F) Same experiment as in (B), but repeated without the challenge (the whole acceleration vector is used here since no lateral force was present). Data are means ± SEM of *n* = 4/23/4 sessions from 4/8/4 mice (HON optostimulation/control/HON optosilencing = C1V1/control/ArchT).

Based on this correlative evidence linking premovement HON activity and anticipatory movements (Fig. 1H), we hypothesized that the premovement HON activation might play a role in regulating the anticipatory movements. To test this hypothesis, we examined whether the anticipatory movements are altered by optogenetic suppression or augmentation of the premovement HON signals, using the previously developed and validated opsins specifically targeted to HONs (7). Laser light application for optogenetic HON silencing or stimulation (in orexin:: ArchT mice and orexin::C1V1 mice, respectively) was precisely restricted to the premovement epoch where premovement HON signals occur (Fig.   2A). Fiberoptically delivered, bilateral laser illumination of the LH profoundly suppressed anticipatory movements in orexin::ArchT mice, and potentiated them in orexin::C1V1 mice [movement examples: Fig. 2(A) middle and bottom; quantification: Fig. 2(B)–(D)]. In control mice without the opsins, the same LH laser illuminations did not evoke such effects (Fig. 2E). These results indicate that the premovement HON activity bidirectionally regulates the anticipatory movements.

Finally, we sought to determine whether premovement HON activation nonspecifically regulates movement acceleration, rather than plays a more specialized role in the control of anticipatory movements. To achieve this, we repeated the bidirectional optogenetic manipulation of premovement HON activity without the force field challenge (Fig. 2F). We quantified the acceleration vector during the same epoch where challenge-anticipatory movements were measured earlier. We found this parameter unaffected by the excitatory opto-manipulations of premovement HON activity (Fig. 2F). Thus, premovement HON activity does not regulate acceleration in all scenarios, but is more specifically involved in anticipatory movements.

## Discussion

Our findings suggest that a physiological role of premovement HON signals is to modulate the initial, anticipatory aspect of skilled movements. This aspect of movement, also known as predictive or feedforward aspect, is thought to be planned *before* movement initiation according to internal models of anticipated challenges, as opposed to feedback/reactive movements that are reactions to challenges occurring “on the fly” *after* the movement is already initiated (1, 9, 10). Predictive control of movement is important, since feedback control can be too slow to produce fast smooth movements (1, 9, 10). Thus, we suggest that premovement HON signals contribute to movement planning, as well as adaptation (11). Since the purpose of premovement HON activity was unknown, this advances our understanding of the role of this core, evolutionarily conserved, neural module in adaptive behavior. About 1:1,000 people suffer from narcolepsy, a debilitating disease associated with HON deficiency; these human patients, as well as HON deficient animals, are strikingly unable to generate appropriate muscle tone in certain challenging situations, leading to postural collapse [reviewed in (12)]. It would be interesting to re-evaluate these motor failures as failures of HON-dependent motor planning, which may diversify existing treatments for these debilitating symptoms, for example by targeting anticipatory postural adjustments. In future work, it would also be important to understand the mechanisms through which HONs regulate anticipatory movements. The HONs’ well-documented ability to stimulate arousal [reviewed in (13)] could be consistent with the present findings, based on the long-proposed link between arousal and learning (14, 15). Premovement HON activation in challenging situations may provide arousal necessary for motor learning, such as internal model updating that is considered key for anticipatory movements (9, 10). Deeper knowledge of the behavioral and cognitive impact of temporally defined HON signals will provide important insights into healthy adaptive behavior.

## Materials and Methods

See Supporting Information where full methods are given.

## Supplementary Material

pgac240_Supplemental_File

## Data Availability

All data are included in the manuscript and/or supporting information.
