# Supplementary material for: Anticipatory countering of motor challenges by premovement activation of orexin neurons
Source: PNAS Nexus. 2022 Oct 25;1(5):pgac240. doi: 10.1093/pnasnexus/pgac240 (PMC9802298; doi:10.1093/pnasnexus/pgac240)
Supplement: pgac240_Supplemental_File [file pgac240_supplemental_file.docx]

**SUPPORTING INFORMATION**

**Materials and Methods**

**Targeting neurons and photons for recordings and optogenetics**

Animal procedures were done in accordance with the Animal Welfare Ordinance (TSchV 455.1) of the Swiss Federal Food Safety and Veterinary Office, and approved by the Zurich Cantonal Veterinary Office. Adult (>8 weeks old) male C57BL/6 mice were used, housed in a reversed 12-h / 12-h light / dark cycle, with experiments done in the dark phase. The targeting of GCaMP6s, C1V1 or ArchT to hypocretin/orexin neurons was performed by LH injection of AAVs, as described and histologically validated in our previous work (1-3). Briefly, for optogenetic experiments, 400 nL of AAV1-hORX.ArchT.TdTomato (1.03*10^13^  GC/mL) or AAV1-hORX.C1V1(t/s).mCherry (> 10^13^ GC/ml) were stereotaxically injected bilaterally into the lateral hypothalamus (coordinates: -1.35, ML: ±0.9, DV: -5.7, 5.4 and -5.1). For neural recording experiments, 200 nL AAV1-hORX.GCaMP6s (2.0*10^13^ GC/mL) at the same coordinates but unilaterally. These manipulations resulted in ArchT, C1V1, or GCaMP6s expression in hypocretin/orexin neurons with high specificity (>96% for each, as validated in by immunostaining in (1-3)). Bilateral (optogenetics) or unilateral (fiber photometry) fiberoptics were implanted into LH for neural control or recording, as previously described (1).

**Sensing and perturbing skilled forelimb movements**

Mice were trained to use their left forelimb to pull a robotic handle to obtain milkshake rewards. The robot (ETH Pattus) comprised a two-degrees-of-freedom robotic handle actuated by motors coupled with rotary encoders (4-6) (Fig. 1A). In all experiments, mice were head-fixed (as described in (7, 8))) within easy reach of the robotic handle, and rewarded (6 µL of milkshake given 500 ms after a correct movement) for pulling 9 mm with maximum lateral deviations of ± 5 mm. After training, this was sufficient for them to engage in pulling sessions of >200 trials over about an hour (one sessions per day performed per mouse, sessions with and without the push challenge were performed by each mouse, in a random order in order to avoid order artefacts). For each trial, a “go cue” (LED) informed the mouse that it could do the pull (Fig. 1B). Handle position was continuously recorded by the robot at 1000 Hz detecting the moment of pull initiation and quantifying the pull acceleration vector before the challenge was imposed later in the pull (Fig. 1A,B). Where indicated, and after the pre-challenge acceleration was recorded, the robot imposed forcefield challenges (lateral pushes) whose average intensity did not change across trials, and which always started at the same location during the pull (0.5 mm in the x direction) and persisted throughout the pull. Kinematic and dynamic data of the robot were acquired in LabView analyzed using MATLAB. The robot provided TTL time-stamps to the neural recording and control systems (described below).

**Acceleration calculations**

For each pull trajectory, the acceleration was computed as the second derivative of x-y position. The lateral (y-direction), pulling direction(x-direction), and magnitude of acceleration (vector component, pull vigor) was then analyzed at the index where the x position first crossed 0.5mm – where the robot can either impose a lateral force field (challenge) or no challenge.

**Recording neural activity**

Fiber photometry recordings of GCaMP6s-expressing hypocretin/orexin neurons were performed using a Doric fiber photometry system as described in (9), and analyzed as described in (10). The neural activity metric, “pre-move HON signal (normalized)” was computed as follows: For each recording session, a convex hull function was fitted to the raw 405nm- excited signal and raw 465 nm-excited signal, then each were divided by this fit to produce a normalized dF/F. The normalized 405nm excited signal was then subtracted from the detrended 465 nm-excited signal. For Figures 1D,E, the signal was fit with a first order polynomial fit from -2 to -1 seconds before movement onset, and then the fit was subtracted from the signal. The signal was then normalized to the movement start value of the baseline trials (26-50) for each session. The pre-movement amplitude was calculated by finding the lowest signal between cue and pull onset, and then subtracted by the value at pull onset. The resulting change in dF/F amplitude was then z scored by dividing each trial from the mean and standard deviation of the baseline 50 trials (Fig. 1F,H,I,J).

**Optostimulation protocols and controls**

Green lasers (532 nm, LaserGlow) were used to bilaterally illuminate the LH via the LH-implanted fiberoptics (11), only during the pre-movement phase (Fig. 2A).

The laser patterns and powers were as follows: 50 Hz, 5ms flashes, 10 mW (orexin::C1V1 optostimulation or controls), continuous 10 mW (orexin::ArchT experiments or controls). Two types of controls were used. “Laser OFF control” (shown in Fig. 2B-D, F) enabled assessment of effects the optogenetic manipulations in the opsin-expressing mice. “Non-opsin control” (Fig. 2E) involved the same LH laser maninpulations as in Fig. 2B, but in mice not expressing opsins. Collectively, these control experiments allowed the impact of specific optomanipulation of hypocretin/orexin neurons to be assessed, while ruling out non-specific effects of laser illumination.

**Statistical analyses**

Data analyses were accomplished using Prism 9 (GraphPad Software Inc, California, USA) and MATLAB 2019b (Mathworks, USA). Where relevant, data were tested for normality using a D’Agostino–Pearson omnibus test, or Kolmogorov–Smirnov test for small sample sizes. To compare interactions within data with repeated measurements, RM ANOVA was used, with multiple comparison tests as described. Data are presented as mean ± SEM unless stated otherwise. P values < 0.05 were considered statistically significant.

**REFERENCES (SUPPORTING INFORMATION)**

1. D. Burdakov, M. M. Karnani, Ultra-sparse Connectivity within the Lateral Hypothalamus. *Current biology : CB* **30**, 4063-4070 e4062 (2020).

2. C. Garau, C. Blomeley, D. Burdakov, Orexin neurons and inhibitory Agrp-->orexin circuits guide spatial exploration in mice. *J Physiol* **598**, 4371-4383 (2020).

3. J. A. Gonzalez *et al.*, Inhibitory Interplay between Orexin Neurons and Eating. *Current Biology* **26**, 2486-2491 (2016).

4. O. Lambercy *et al.*, Sub-processes of motor learning revealed by a robotic manipulandum for rodents. *Behavioural brain research* **278**, 569-576 (2015).

5. B. Vigaru *et al.*, A small-scale robotic manipulandum for motor training in stroke rats. *IEEE Int Conf Rehabil Robot* **2011**, 5975349 (2011).

6. B. C. Vigaru *et al.*, A robotic platform to assess, guide and perturb rat forelimb movements. *IEEE Trans Neural Syst Rehabil Eng* **21**, 796-805 (2013).

7. N. Grujic, J. Brus, D. Burdakov, R. Polania, Rational inattention in mice. *Sci Adv* **8**, eabj8935 (2022).

8. P. P. Vidal, L. Degallaix, P. Josset, J. P. Gasc, K. E. Cullen, Postural and locomotor control in normal and vestibularly deficient mice. *J Physiol* **559**, 625-638 (2004).

9. H. T. Li, D. C. Donegan, D. Peleg-Raibstein, D. Burdakov, Hypothalamic deep brain stimulation as a strategy to manage anxiety disorders. *Proceedings of the National Academy of Sciences of the United States of America* **119**, e2113518119 (2022).

10. P. Viskaitis *et al.*, Ingested non-essential amino acids recruit brain orexin cells to suppress eating in mice. *Current biology : CB* **32**, 1812-1821 e1814 (2022).

11. M. M. Karnani *et al.*, Role of spontaneous and sensory orexin network dynamics in rapid locomotion initiation. *Progress in Neurobiology* **187**, 101771 (2020).
